# The core sequence of PIF competes for insulin/amyloid β in insulin degrading enzyme: potential treatment for Alzheimer's disease

**DOI:** 10.18632/oncotarget.26057

**Published:** 2018-09-21

**Authors:** Soren Hayrabedyan, Krassimira Todorova, Marialuigia Spinelli, Eytan R. Barnea, Martin Mueller

**Affiliations:** ^1^ Institute of Biology and Immunology of Reproduction, Bulgarian Academy of Sciences, Laboratory of Reproductive OMICs Technologies, Sofia, Bulgaria; ^2^ Department of Obstetrics and Gynecology, University Hospital Bern, University of Bern, Bern, Switzerland; ^3^ Society for The Investigation of Early Pregnancy (SIEP), New York, NY, USA; ^4^ BioIncept, New York, NY, USA; ^5^ Department of Obstetrics, Gynecology and Reproductive Sciences, Yale University School of Medicine, New Haven, CT, USA; ^6^ Department of Paediatrics, School for Mental Health and Neuroscience, Maastricht University, Maastricht, The Netherlands

**Keywords:** PreImplantation factor (PIF), Alzheimer's disease, insulin degrading enzyme (IDE)

## Abstract

The central pathological feature of Alzheimer's disease (AD) is the sequential proteolytic processing of amyloid precursor protein (APP) to amyloid-β peptides (Aβ) agglomeration. The clearance of Aβ may be induced by the large zinc-binding protease insulin degrading enzyme (IDE). IDE is the common link between AD and Type II diabetes as insulin is an IDE target as well. Not surprisingly, the search for safe and effective drugs modulating IDE is ongoing. A new pregnancy derived peptide, PreImplantation Factor (PIF), inhibits neuro-inflammation and crosses the blood-brain-barrier. Importantly, we report that the (R_3_I_4_K_5_P_6_) core sequence of the PIF peptide modulates IDE function and results in decreased Aβ agglomeration in neuronal cells. Using bioinformatics we show that PIF binds to the IDE complex and sterically competes for the same place as insulin or Aβ. The predicted RIKP sequence and especially the specific I^4^ and P^6^ amino acids are essential for hydrophobic interactions with the IDE complex. In terms of potential AD treatment, PIF was successfully tested in neurodegenerative animal models of perinatal brain injury and experimental autoimmune encephalitis. Importantly, sPIF received a FDA Fast Track Approval and orphan drug designation for first-in-human trial in autoimmunity.

## INTRODUCTION

Sequential proteolytic processing of amyloid precursor protein (APP) leads to amyloid-β peptides (Aβ) agglomeration, the central pathological feature of Alzheimer's disease (AD) [1, 2]. Development of effective and safe disease modifying treatments that directly target AD pathology is a priority especially as the drugs currently available for AD only manage symptoms and do not target Amyloid plaques [3]. A potential target is the large zinc-binding protease insulin degrading enzyme (IDE) as IDE is involved in clearance of insulin and Aβ [2, 4]. Multiple authors postulate that IDE is the common link between AD and Type II diabetes [2, 5, 6]. For example, decreased IDE expression was reported in AD patients [7] and IDE polymorphisms were associated with impaired insulin metabolism [8]. Chemical IDE inhibitors improve insulin activity [9] while IDE activators regulate Aβ formation in neurons [2, 10]. Finally, the increase in a given IDE variant reduces circulating Aβ levels, while other variants promote the disease [11, 12]. Together, targeting IDE is an attractive strategy to prevent AD and type II diabetes but safe and potent drugs are currently lacking [2].

Recently, a new pregnancy derived peptide emerged in the arena of neurotherapeutics. PreImplantation Factor (PIF) can be detected in the maternal circulation during pregnancy [13, 14] and its presence has been correlated with live birth [14–16]. PIF has been implicated in promoting embryo implantation through modulating maternal immune tolerance [14, 17–19]. Consistent with the immunomodulatory function, a synthetic PIF analog (sPIF) was able to reverse and prevent paralysis and restore myelination through inhibiting neuro-inflammation in murine models of experimental autoimmune encephalomyelitis [20, 21]. The neuroprotective property of sPIF was further underscored by its ability to mitigate neuronal loss and microglial activation in murine model of immature brain injury [22, 23]. Here we report that the (R_3_I_4_K_5_P_6_) core sequence of PIF modulates IDE function and results in decreased Aβ agglomeration in neuronal cells. This is in line with PIF`s pleiotropic function both in pregnancy and in non-pregnant setting [24]. Additionally, preclinical studies identified a synthetic analog of PIF (sPIF) as an effective drug in autoimmune diabetes [25], atherosclerosis [26], graft versus host disease [27], and radiation induced pathology [28]. In terms of potential AD treatment, sPIF crosses the blood-brain barrier and was successfully tested in neurodegenerative animal models of perinatal brain injury [22, 23] and experimental autoimmune encephalitis [20, 21]. Importantly, sPIF received a FDA Fast Track Approval for first in human trial in autommune hepatitis.

## RESULTS

### PIF reduces Aβ in IDE dependent manner

**Figure 1:**
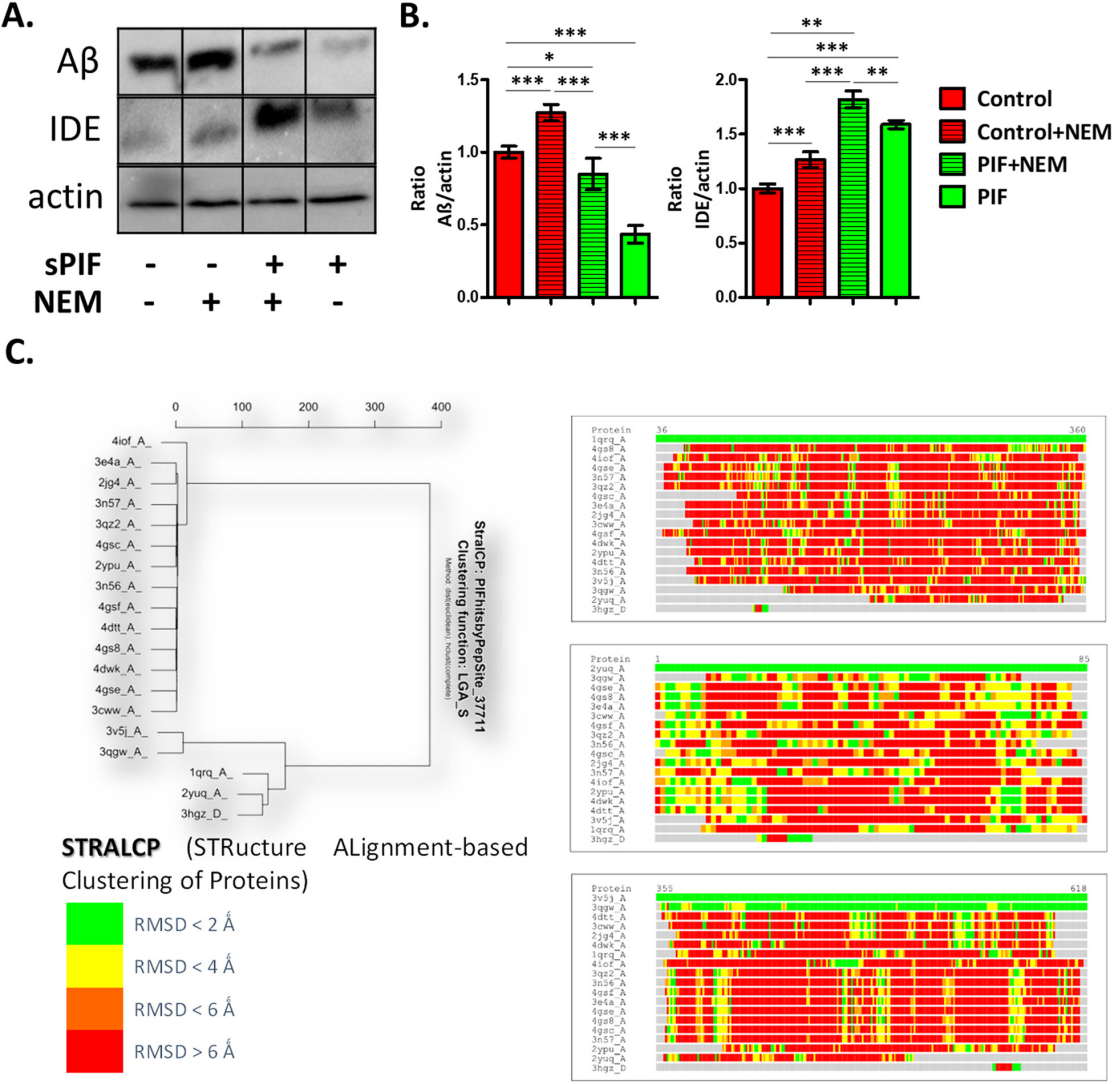
PIF reduces Aβ formation in IDE dependent manner and targets distinct protein families **(A)** Representative Western Blots of Aβ and IDE in neuronal cells after APP transfection. **(B)** sPIF reduces Aβ formation in IDE dependent manner. **(C)** Identified regions of structural similarity within the set of protein structures by STRALCP. Clustering of structurally conserved fragments (*left*). STRALCP identified structural similarity between selected “*reference*” structure and other analyzed structures, represented as colored bars, based on C_alpha_ - C_alpha_ distance deviation at each position between the reference (top bar) and other structures (*right*). The colors indicate RMSD between aligned residues, ranging from green (below 2Å), yellow (below 4Å), orange (below 6Å), to red (above 6Å). STRALCP: STRucture ALignment-based Clustering of Proteins; RMSD: Room mean square distances; Aβ: Amyloid Beta; IDE: Insulin degrading enzyme; APP: Amyloid precursor protein; sPIF: synthetic PreImplantation Factor; ^*^ p<0.05; ^**^ p<0.01; ^***^ p<0.001 (ANOVA followed by two tail t test). *In-vitro* experiment results represent at least three independent experiments.

In order to evaluate PIF-IDE interactions in the context of AD we stably transfected neuronal cells (N2a) cells with human APP695 (APP-N2a) to increase endogenous APP levels first [29]. Notably, this setup represents an *in-vitro* AD model as sequential proteolytic processing of APP results in Aβ formation and Aβ is an IDE target. We treated cells with sPIF and indeed such treatment decreased Aβ formation while increasing IDE levels significantly (Figure 1A and 1B compare red and green bars). Importantly, in the presence of the IDE inhibitor N-ethylmaleimide (NEM) abolished the sPIF induced effects confirming PIF-IDE interaction.

### The RIKP sequence participates in PIF-IDE interaction

**Figure 2:**
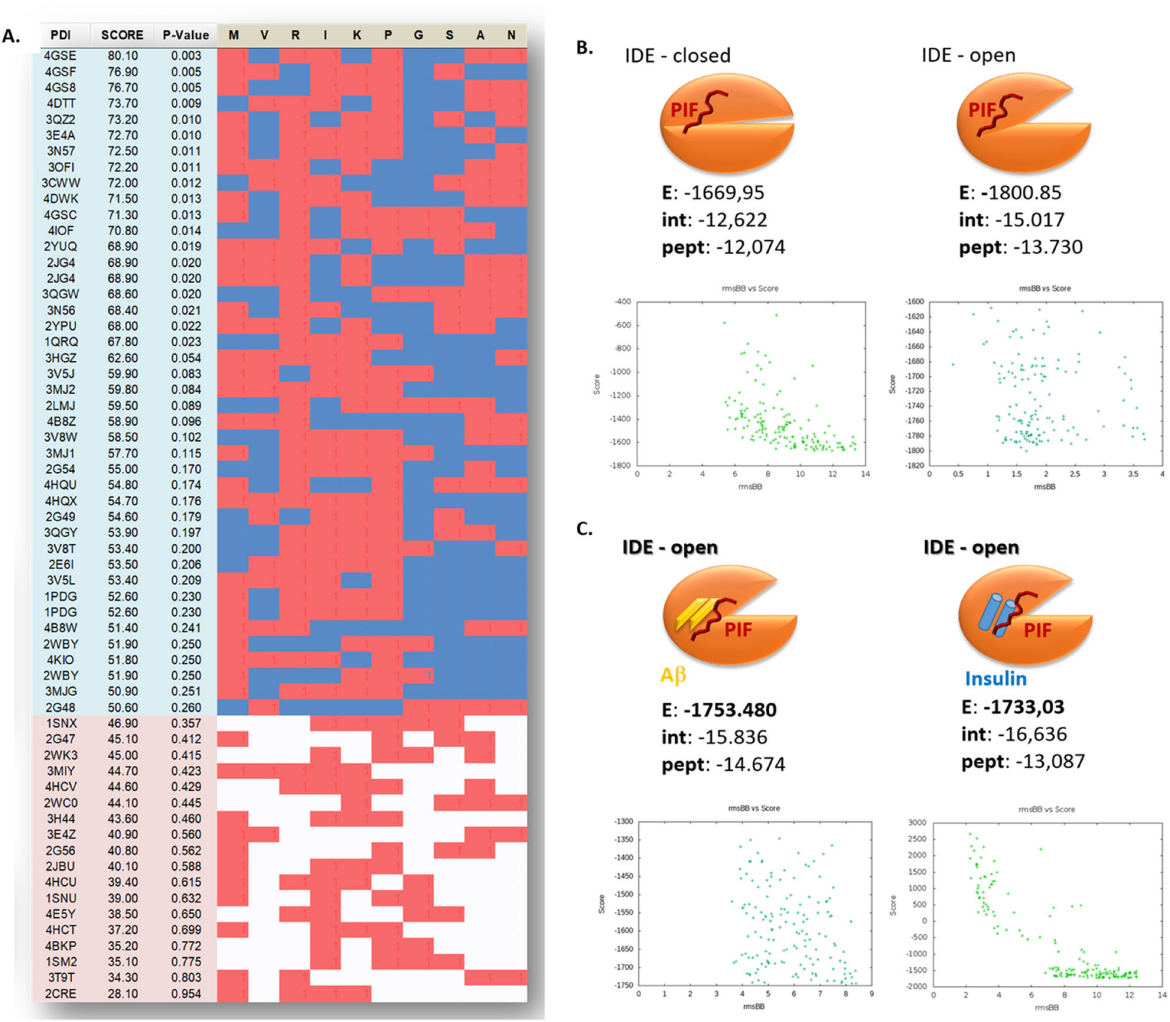
The Arg-Ile-Lys-Pro (RIKP) sequence participates in the PIF targets interaction and sterically competes for the same binding site as insulin or Aβ **(A)** Prediction of PIF peptide:protein binding interface with putative PIF:target binding residues interaction of ProtoArray^®^ true positive and negative. We determined the statistical probability of important residues and identified the M^*^RIKP^****^ sequence to be most likely binding pattern. Red squares are for a hit, blue or white are for a miss while blue is in the region of positive hits, and the white is in the region of negative hits. **(B** and **C)** Cartoons representing the flexible in silico peptide-protein docking (FlexPepSite) of PIF to IDE. (B) PIF interacts with IDE (in both open and closed conformation) but has the higher Energy in open conformation. (C) PIF binds to IDE (open conformation) in combination with Aβ or Insulin with lesser Energy than to IDE alone. Energy is measured in Rosetta Energy Unit (REU). Aβ: Amyloid Beta; IDE: Insulin degrading enzyme; PIF: PreImplantation Factor.

To further dissect the PIF-IDE interactions we retrieved molecular models corresponding to the 10 PIF targets obtained by experimental methods (ProteoArray and proteomics) and their homologous protein family members. Using Protein Data Bank (PDB) we extracted about 200 crystallography generated PDBs corresponding to the positive protein hits and more than 2500 crystallography generated PDBs corresponding to the negative protein hits and discarded the redundant ones to 60 and 2369 accordingly (*see* Supplementary Table 1). Using STRALCP (STRucture ALignment-based Clustering of Proteins) algorithm we identified multiple distinct protein families sharing a common structural “pattern” (Figure 1C). We determined the statistical probability of important residues resulting in the identification of M^*^RIKP^****^ amino sequence with the predicted small sequence (RIKP) likely to participate in peptide/protein interactions. PepSite had 95.24% true positive detection rate in PDBs corresponding to PIF positive targets identified using ProteoArray, while the PDBs corresponding to the negative targets available in the rest of the protein array had 40.33% false positive score (Figure 2A) [30]. Indeed, R^3^ and K^5^ seem to be most promising positive hits followed by the A^9^ and N^10^. Together, we identified the RIKP sequence as the most commonly participating in potential PIF-IDE interaction matching the R^3*^K^5****^N^10^ signature.

### PIF sterically competes for the same binding site as insulin or Aβ

**Figure 3:**
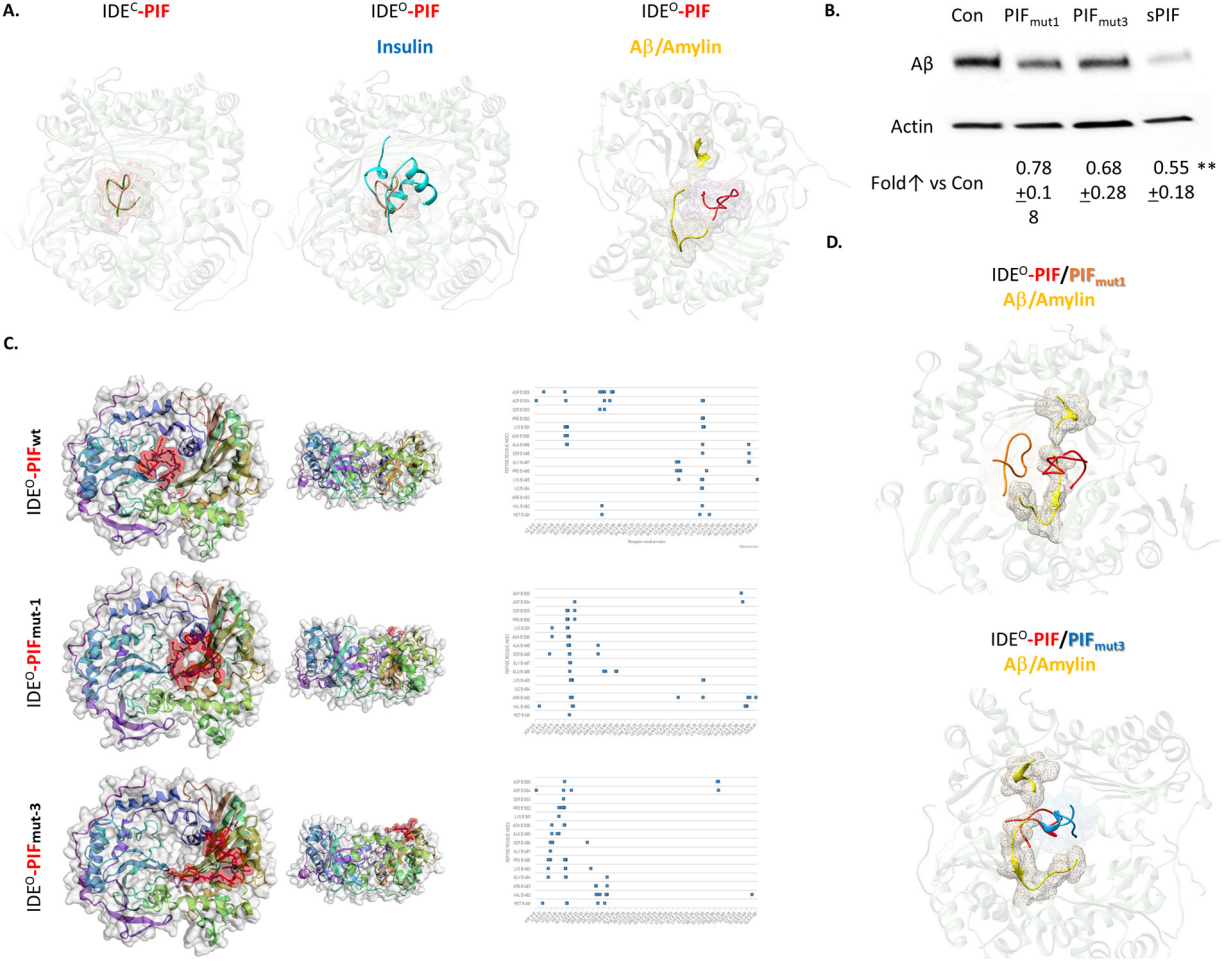
PIF competes with Insulin and Aβ for binding to IDE, but it binds to distinct sites **(A)** Flexible peptide*in silico* docking of PIF to crystallography models of IDE in a complex with Insulin or with Aβ/Amylin, based on predicted binding site. Pink mesh shows PIF (in red) binding region. Blue represents Insulin and yellow Aβ or Amylin. **(B)** Representative Western Blots of Aβ in neuronal cells, after APP transfection, treated with sPIF and PIF_mut1 and 3_. **(C)** CABS Dock blind *in silico* docking of flexible PIF peptide (red) to IDE based on molecular dynamics. Binding interface is determined by interacting PIF AA residues with IDE, defined as PIF AA versus IDE AA. This is reflected as a shift of the putative binding interface (distance cutoff 4.5 Å) from deeper to more superficial AA residues in the PIF binding groove. This algorithm does not use PepSite2 predicted binding spots but rather scans IDE for binding affinity blindly. The docking/binding models visually confirm that the PIF_mut1_ is shifted out of the wild type binding pocket, while the PIF_mut3_ is completely removed from the pocket and rather binds by different set of IDE AA residues. **(D)** Flexible peptide*in silico* docking of PIF and PIF_mut1 and 3_ to crystallography models of IDE^O^ in a complex with Aβ/Amylin, based on predicted binding site. Red (PIF), orange (PIF_mut1_), and blue (PIF_mut3_) meshes show binding regions in the presence of Aβ/Amylin (yellow).Aβ: Amyloid Beta; IDE: Insulin degrading enzyme; IDE^C / O^: Insulin degrading enzyme in closed or open confirmation; PIF: PreImplantation Factor; Con: Control. ^**^p<0.01 (ANOVA followed by two tail t test). *In-vitro* experiment results represent at least three independent experiments. two-tailed Student's t-test and results are presented mean + SD.

Given the predicted RIKP signature of PIF, we aimed to identify the potential and specific amino acid interaction sites with IDE next. Notably, IDE has two principal forms [31] with closed form (IDE^C^) having minimal while the open form (IDE^O^) having high catalytic activity [32]. IDE is activated by ATP to degrade short peptides such as Aβ or insulin by a change in enzyme conformation to IDE^O^ [33]. We predicted the putative binding sites of PIF using PepSite2 server and then the *de novo* modeled PIF was docked to crystallographic models of IDE in open ligand bound and closed ligand free state (FlexPepDock flexible docking server). Indeed, the PIF-IDE complex acquires its highest energy gain when PIF is bound to IDE^O^ compared to IDE^C^ (Figure 2B). Importantly, PIF-IDE^O^ complex forms high affinity bond in Aβ and insulin presence (Figure 2C). The distance of PIF from the binding pocket increases when Insulin is present in order to maintain stable molecular complex (Figure 2C and 3A). This suggests that PIF sterically competes for the same place as insulin. We can replicate this in case of excessive Aβ bound to IDE as well, where similarly PIF and Aβ/Amylin is bound to the very same pocket (Figure 2C and 3A). Interestingly, PIF binding Energy is even stronger when open IDE^O^ conformation has already attached Aβ or Amylin. Stabilization of the molecular complex occurs only when PIF is repulsed back to 4 Å distance. Theoretically, PIF is less prone to bind IDE-Aβ than IDE-Insulin, but still it forms high affinity bond despite Aβ presence, when compared to free IDE^C^ conformation. Together, PIF binds to the IDE^O^ complex and sterically competes for the same place as insulin or Aβ.

### The RIKP sequence (I^4^ and P^6^) are essential for PIF-IDE interaction

**Figure 4:**
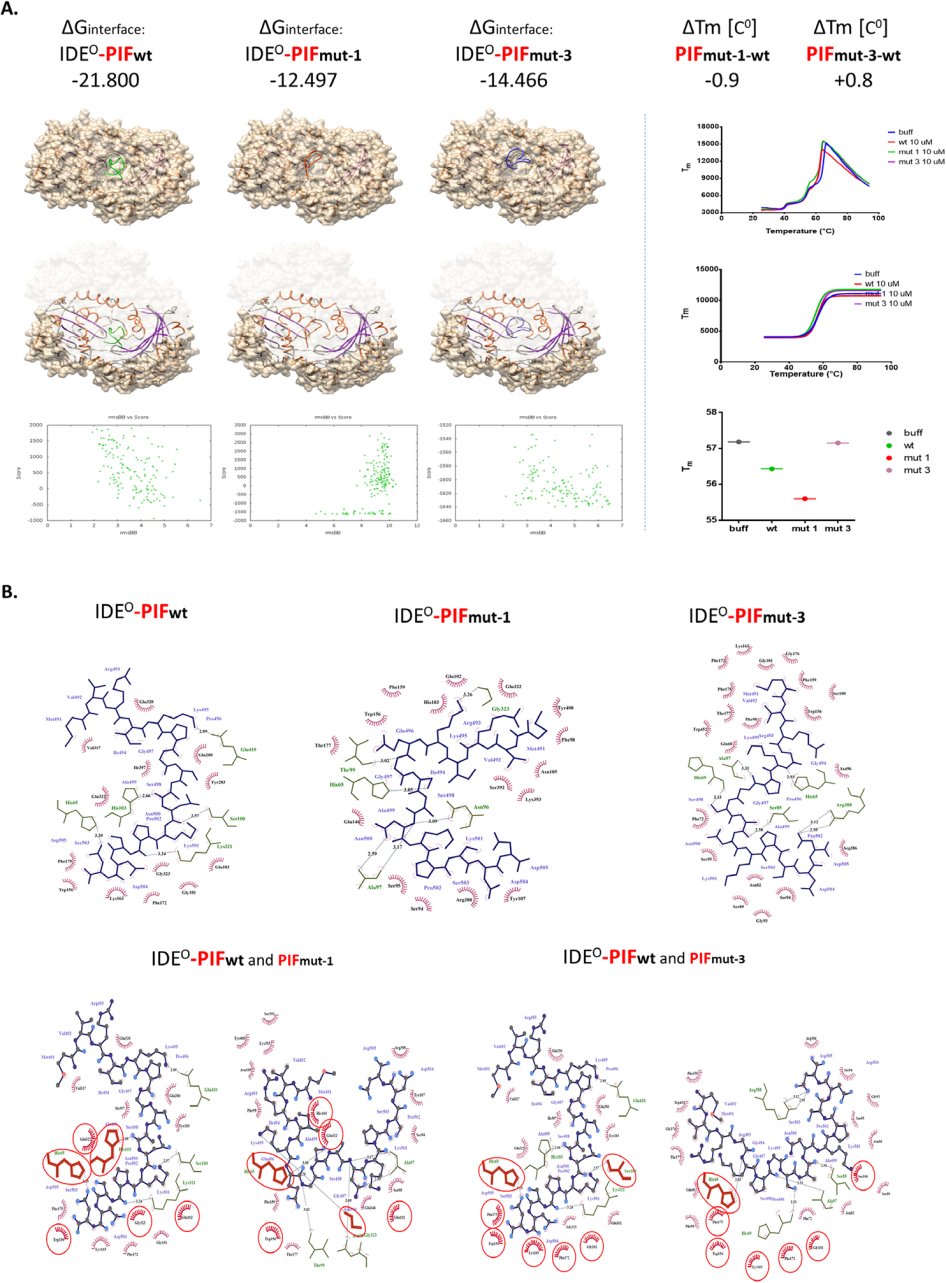
Flexible *in silico* docking of PIF_wt_ versus PIF_mut1_ and PIF_mut3_ **(A)** The binding Energies correspond to binding affinity and are in line with Differential Scanning Fluorimetry data (*right panel*), demonstrating that PIF_mut1_ has a reduced binding affinity and thermal shift (ΔTm). This results in a weaker PIF_mut1_ binding to IDE (hence lower stabilization of the IDE energy). PIF_mut3_ has also lower binding affinity to the site of PIF_wt_ binding, but its ligand-receptor interaction validation demonstrated slight positive melting shift, and increase of IDE stabilization energy, suggesting increased affinity to IDE. Binding models RMSD suggest displacement of PIF from its natively predicted binding site upon mutagenesis. Similarly, Chimera visualizations of the docking models confirm this location shift out of the groove (*left cartoons*). **(B)** Planar schemes of PIF_wt/mut-1/mut-3_-IDE interface bindings. In the upper panels, we demonstrate that the PIF_wt_ C-terminus binds Gly101 or Ser100. When mutated (loss of Pro6: PIF_mut-1_) PIF binding sites change such as Ala97 or Gly323. Together, this results in a shift in the binding site upwards of the groove (*left panels*). The loss of Ile4 (PIF_mut-3_) causes a similar shift in the IDE binding residues, but with increasing the probability of binding to Ala97, His65, or Pro171. Binding mechanics of PIF to IDE suggests that PIF_mut-1_ disrupts the interface by losing hydrogen bonds, while PIF_mut-3_ although predicted as lowering affinity, gains more hydrophobic interactions. As demonstrated in the lower panels, the binding mechanics to IDE includes both hydrogen bonds (lines with distance in Å) and hydrophobic contacts (red “eyelash”). With blue lines—PIF chemical bonds, in green—IDE residues participating in the interface, all residues shared between PIF_wt_ and PIF_mut-1/3_ are circled in red. The models confirm that PIF_mut-1_ has fewer hydrogen bonds and hydrophobic interacting residues, while PIF_mut-3_ displays increasing hydrophobic interacting residues. Aβ: Amyloid Beta; IDE: Insulin degrading enzyme; IDE^C / O^: Insulin degrading enzyme in closed or open confirmation; PIF: PreImplantation Factor.

To further dissect the importance of individual amino acid residues in PIF sequence, we generated putative PIF mutants (PIF_mut_) using BeatMusic server for in silico mutagenesis (Supplementary Figure 1). For further analysis we selected PIF_mut1_ (mutation P_6_ to E_6_) which was specific for the active form of IDE and having the highest change in Energy of binding (Figure 3C, 4A). The second mutant was PIF_mut3_ (I_4_ to G_4_) which was predicted to disrupt the binding with chain B of active IDE while affecting both chains in the IDE closed conformation (Figure 3C, 4A). In order to provide further evidence that PIF unique amino acid structure is responsible for specific PIF-IDE interactions we stably transfected neuronal cells with human APP695 (APP-N2a) again. Indeed, PIF_mut1 and 3_ treatments did not decrease Aβ formation as efficient as sPIF (Figure 3B). We further re-docked the *in silico* estimated PIF mutants (PIF_mut1_ and PIF_mut3_). The docking/binding models visually confirmed that PIF_mut1_ (P_6_ to E_6_) is shifted out of the wild type binding pocket (distance cutoff 4.5 Å), while PIF_mut3_ (I_4_ to G_4_) is completely removed from the pocket and rather binds different set of IDE AA residues (Figure 3C and 3D). Generally, both mutants have lower probability and higher distance from IDE grove and less favorable energy profile to bind to IDE^O^ in presence of AD as shown using *in silico* docking binding interface determined by interacting residues form PIF and IDE (Figure 4A).

### The I^4^ and P^6^ amino acids participate in hydrophobic interactions of the PIF-IDE complex

Finally, we determined to examine the structure-function relationship and exposed IDE in cell free environment (recombinant enzyme) to synthetic PIF. We used Differential Scanning Fluorimetry (DSF) to identify low-molecular-weight ligands that bind and stabilize purified proteins. Notably, we used a fluorescent dye-based probe that preferentially binds the hydrophobic regions of a protein, which are increasingly exposed during protein denaturation. Therefore, a ligand bound to a protein, e.g. to its active site, has the propensity to increase its thermal stability (and hence it's Tm) through newly formed ligand-protein interactions allowing prospective binding ligand-affinity assessment [34]. We detected only PIF_wt_ to bind effectively to the protein target (Figure 4A right panel) while the PIF_mut1_ showed reduced binding affinity and decreased ΔT_m_. In contrast, we detected minimally increased affinity of binding measured as positive ΔT_m_in PIF_mut3_. In order to determine the specific reason why the PIF_mutants_ displayed reduced ΔT_m_ or disrupted ΔG_interface_, we generated planar schemes of PIF-IDE interface binding (Figure 4B). Interestingly, we detected loss of multiple hydrogen bonds and hydrophobic interactions in PIF_mut1_ compared to PIF_wt._. Although PIF_mut-3_ lost as many hydrogen bonds as PIF_mut-1_, we detected increased hydrophobic interacting residues when “sliding out” of the natural groove (Figure 4A and 4B). PIF_mutants_ predominantly bind closer to N-terminus amino acid residues of the IDE^O^ chain. The PIF_wt_ C-terminus binds Gly101 or Ser100 (Figure 4B). When mutated (loss of Pro6: PIF_mut-1_) PIF binding sites change such as Ala97 or Gly323. Together, this results in a shift in the binding site upwards of the groove. The loss of Ile4 (PIF_mut-3_) causes a similar shift in the IDE binding residues, but with increasing the probability of binding to Ala97, His65, or Pro171. Together, the predicted RIKP sequence and especially the specific I^4^ and P^6^ amino acid location are essential to for hydrophobic interactions with the IDE^O^ complex.

## DISCUSSION

We provide evidence that PIF multi-targeting interaction with IDE is exerted through the (R_3_I_4_K_5_P_6_) core sequence. This is of importance given PIF as an evolutionary conserved peptide exerting pleiotropic functions such as immune regulatory and neuroprotective properties [24]. The amino acid core sequence itself will not explain the binding to different targets with their associated effects. However, it provides evidence that PIF induces peptide/protein interactions that may be structurally and functionally unrelated but they share local structural similarities. For example, the binding region of PIF to IDE is highly similar to that of protein disulfide isomerases (PDI) and heat shock protein 70 (HSP70), which are both PIF targets [30]. Therefore, the flexible PIF peptide binds to specific target protein “pockets” and modulates their interaction and specific function. The specific RIKP structure is of importance as the docking analysis comparing PIF_wt_ to PIF_mutants_ displayed (Figures 3 and 4). Further, PIF may rather (or in addition) stabilize the IDE in the open conformation, and make its active pocket more accessible to substrates. At least four factors contribute to the unique mechanism of substrate recognition by IDE [4].

One factor contributing to the substrate recognition is the proper anchoring of the cleavage site in the catalytic cleft. As seen in the Binding Interface Contact Map (Figure 3B, right panel), PIF_mutants_ predominantly bind closer to N-terminus amino acid residues of the IDE^O^ chain. The PIF_wt_ C-terminus will bind Gly101 and Ser100 (Figure 4B). When mutated (loss of Pro6: PIF_mut-1_) or loss of Ile4 (PIF_mut-3_), PIF targets change and results in a shift in the binding site upwards of the groove. Therefore, the PIF core sequence is one factor determining PIF-IDE interaction. The other factor is the IDE binding pocket. The PIF-IDE effects are induced due to competition for the same IDE pocket binding site as insulin or Aβ. The factor determining which peptide will bind to IDE are the hydrogen bonds (Figure 4B and 4C). PIF_wt_ do not have significant positive charges at the C-termini and therefore avoids the charge repulsion from IDE^O^. This is especially true in comparison to other IDE-substrate structures such as insulin, Aβ, or Amylin (Figure 3A) where PIF sterically competes for the same binding site. Multiple other substrates such as brain natriuretic peptide, glucagon-like peptide and insulin growth factor I have many positively charged residues at their C termini and are poor IDE substrates [4]. Therefore, PIF effects would be less evident. By contrast, substrates, which lack positive charges at their C termini are excellent IDE substrates and could represent a potential PIF target. These include the related hormones atrial natriuretic peptide, glucagon, and IGF-II but the PIF potential interactions with these peptides are beyond the scope of this manuscript.

At last, the size is an important contributor. The IDE catalytic chamber is large enough to accommodate only relatively small peptides (estimated to be <50 amino acids) [4]. As an M16A member of the Zn^2+^-metalloprotease family, IDE has a buried catalytic site in the structure and the closed–open conformational switch kinetically controls access to this chamber. Therefore, the self-oligomerization may allosterically regulate the catalytic activity of IDE and represents the advantage of a small molecule like PIF. Not surprisingly, a large number of chemical modulators of IDE activity such as chelators, divalent cations, thiol-blocking agents, curcumin, docosahexaenoic acid, or sevoflurane were identified. However, their use as therapeutic agents is limited due to low potency, non-selectivity and/or highly toxicity [2]. Importantly, the IDE dependent reduction of Aβ agglomeration in neuronal cells (Figure 1A and 1B) by PIF is in line with previous neuroprotective effects [24], which supports PIF use in AD.

The use of PIF in AD is supported by the fact that sPIF crosses the blood-brain barrier and was successfully tested in neurodegenerative clinically relevant models of perinatal brain injury [22, 23] and experimental autoimmune encephalitis [20, 21]. We are not providing here *in-vivo* data supporting the use of PIF in AD, which is a limitation of our study. Animal models of AD are currently being prepared but are beyond the scope of this manuscript. However, sPIF received a FDA Fast-Track Approval for first-in-human Phase I trial and orphan drug designation for autoimmunity, which was successfully completed [35]. Such observations together with the current evidence support PIF's potential protective role in AD or type II diabetes, where IDE plays a major role.

## MATERIALS AND METHODS

### PIF peptide synthesis

Synthetic PIF (MVRIKPGSANKPSDD), scrambled (inactive PIF) GRVDPSNKSMPKDIA and mutated PIF I^4^ to G^4^ (MVR**G**KPGSANKPSDD) or P^6^ to E^6^ (MVRIK**E**GSANKPSDD) were produced using solid-phase peptide synthesis (Peptide Synthesizer, Applied Biosystems, Foster City, CA, USA) employing Fmoc (9-fluorenylmethoxycarbonyl) chemistry. Final purification was carried out by reverse-phase high-pressure liquid chromatography (HPLC), and peptide identity was verified by mass spectrometry. Alexafluor 647-PIF conjugate was generated (Bio-Synthesis, Inc., Lewisville, TX, USA). N-Ethylmaleimide was obtained from Sigma-Aldrich, St Louis MO, USA.

### STRALCP (STRucture ALignment-based clustering of proteins)

Protein classification is essential for proper protein's structure, function and interaction with other proteins prediction. Although sequence similarities correspond to structure similarities most of the time, structural similarity, however, does not necessarily correspond to sequence similarity [36]. Thus, structural comparison and classification allows for structure and function prediction for uncharacterized proteins, and in our case finding common ground for multiple targets of same short PIF peptide ligand. STRALCP identifies regions of structural similarity within a given set of protein structures and uses those regions for hierarchical clustering. STRALCP generates detailed information about global and local similarities between pairs of protein structures, identifies fragments (spans) that are structurally conserved among proteins, and uses these spans to group the structures accordingly. Color coded bars based on structural similarity and cluster maps between the referent PDB structure and all others are produced by the server. We used the PDB IDs from PDB databank corresponding to the previously identified by ProteoArray™ targets of PIF. As each PIF protein target had multiple crystallography PBDs corresponding to it, a preliminary redundancy discarding step was carried out first. The parameters fed to the Local-Global Alignment (LGA) scoring algorithm were option “-4-sia” (searching for residue-residue correspondence, where the best superposition is calculated completely ignoring sequence relationship between reference and compared proteins, and the suitable amino acid correspondence (structural alignment) is reported) [36]. Thus, STRALCP lead to understanding the structural similarity of common PIF targets and derive conclusions for shared modes of PIF actions and its interaction with other molecules. We used the following web server at http://proteinmodel.org/AS2TS/STRALCP/ for selecting protein structures, calculating structurally conserved regions and performing automated clustering [37].

### *In silico* modeling of the PIF binding to protein targets

PepSite 2 server (http://pepsite2.russelllab.org/) was used for *in silico* prediction of PIF peptide binding sites on protein surfaces, referred hereon as PepSite only. As PepSite server is limited to 10 amino acids (aa) for peptide sequence, only the first 10 aa were used, as it was shown previously that the first 9 aa are shared between the two biologically active PIF forms, both having the same actions [38]. PepSite was used in two different manners: 1). in an automated workflow governed by the Taverna bioinformatics workflow framework, to query all PDBs corresponding to PIF positive and negative targets (from ProteoArray™) using REST service, taking into account the top scored replies (PDB selection is described in STARLCP method). Then the ranked tuples of PIF peptide binding sites, PepSite binding Score and p-value of binding prediction, were all used to categorize the different positive and negative targets and generate enrichment statistics using positional partial cumulative probability scores. Secondly, PepSite was also used in in silico validation studies, where PIF predicted position (defined as geometric centers of amino acid residues) of binding to IDE was used to further flexible dock PIF_wt_ and PIF_mutants_ to IDE open and closed forms using Rossetta FlexPepDock (see below). In PepSite predicted binding to positive/negative PIF targets, p-Value defined the probability of erroneous binding. Additionally, the ranking of the targets and the Partial Cumulative Probability were calculated using 1-p-Value Probability of true binding, divided to 10 (as the number of AA in PIF sequence used by PepSite) and also divided by the number of PDBs in each tested group.

### Rosetta FlexPepDock server

Rosetta FlexPepDock server (http://flexpepdock.furmanlab.cs.huji.ac.il/) for high-resolution peptide docking was used as well. We used as input a PDB file consisting of the PIF protein target as a first chain: Insulin degradation enzyme, PDB models 2WBY (crystal structure of IDE in complex with Insulin) and 2JG4 (crystal structure of substrate-free IDE in its closed conformation) and PIF peptide conformation predicted *ab initio* using the Pep-Fold server (http://bioserv.rpbs.univ-paris-diderot.fr/PEP-FOLD/) as a second chain. The peptide location was derived from the binding prediction obtained using PepSite 2. Notably, FlexPepDock allows full flexibility to the peptide and side-chain flexibility to the target protein. Thus, it provides accurate refinement of the peptide structure, starting from up to 5.5A RMSD (root-mean-square deviation (RMSD) is the measure of the average distance between the atoms (usually the backbone atoms) of superimposed proteins) of the native conformation [39].

All protein image rendering and the multiple sequence alignment (MSA) following multiple protein structural alignment (superposition) was performed using Chimera v.1.8c (UCSD, US). A local Smith-Waterman alignment algorithm, BLOSUM 35 matrix, 80-90% secondary structure score inclusion, and 3 A iterative matching, were all used for the structural alignments. MSA was performed using superposition and circular permutation, with residue-residue distance cutoff of 5Å. MultiProt server (http://bioinfo3d.cs.tau.ac.il/MultiProt/) was used for partial multiple alignment of the protein targets identified by ProtoArray^®^, using algorithm for local multiple structural alignment of common geometrical cores [40].

### CABS-dock server

CABS-dock serverperforms blind flexible protein-peptide docking by searching for the binding site, allowing for full flexibility of the peptide and small fluctuations of the receptor backbone. The blind docking generates peptide model and carries out molecular dynamics of the receptor while searching for binding space. First random structures of the peptide are generated and randomly placed on the surface of the sphere centered at the receptor's geometrical center then Replica Exchange Monte Carlo molecular dynamics with 10 replicas uniformly spread on the temperature scale is utilized. Additionally, the temperatures of the replicas constantly decrease as the simulation proceeds to end on the bottom of the energy minima. On output the procedure produces 10 trajectories (one for each replica), each consisting of 1000 time-stamped simulation snapshots for a combined total of 10,000 models. During the simulation, the receptor molecule is kept in near-native conformations by a set of distance restraints binding pairs of C-alpha atoms. Selection of the final representative models follows a two-step procedure: Initial filtering of the 10 trajectories, excluding all unbound states. Selected models (1000 in total) are clustered together in the k-medoids procedure down to 10 final models. This algorithm allows for selection of docking models without prior knowledge of the peptide binding site. Additionally, regardless of the rigid docking approaches, a molecular dynamics ensures fluctuation of the receptor and full plasticity of the binding ligand [41]. CABS-dock web server for the flexible docking of peptides to proteins without prior knowledge of the binding site [41].

### LigPlot+

LigPlot+ was used for automatic generation of schematic diagrams of protein-ligand interactions for a given ligand in a PDB file. The software is based on LIGPLOT and DIMPLOT and allows for flattened view of the PIF interaction upon *in silico* docking. Additionally, related LIGPLOTs superposing highlight similarities and differences between related proteins binding the same/similar ligand, or the same/similar ligand binding to different proteins. LigPlot+ attempts, as best it can, to place residues in the new plot on top of the equivalent residues in the old one.

### Differential scanning fluorimetry (DSF)

Differential scanning fluorimetry (DSF) is a method used to identify low-molecular-weight ligands that bind and stabilize purified proteins. The temperature at which a protein unfolds is measured by an increase in the fluorescence of a dye with affinity for hydrophobic parts of the protein, which are exposed as the protein unfolds. A simple fitting procedure allows quick calculation of the transition midpoint; the difference in the temperature of this midpoint in the presence and absence of ligand is related to the binding affinity of the small molecule, which can be a low-molecular-weight compound, a peptide or a nucleic acid. DSF monitors thermal unfolding of proteins in the presence of a fluorescent dye and is typically performed by using a real-time PCR instrument [34]. The thermal shift assays were conducted on an Mx3005P from Stratagene, originally designed for real-time quantitative PCR. The system contains a heating/cooling device for accurate temperature control, a 488 nm argon-ion laser excitation source, a dual-axis synchronous scanning head to distribute the excitation light to the 96 wells of a microplate, a spectrograph, and a charge-coupled device camera for the fluorescence detection from 500 to 600 nm. It permits then the simultaneous imaging of the fluorescence changes in the microplate recorded using MXP software (Stratagene, La Jolla, CA). DSF analysis protocol followed the Guidelines issued by the Structural Genomics Consortium at Oxford University for Compound Screening [34].

Thermal stability measurements were recorded in 96-well PCR plates covered with optical film, using the described above PCR thermal cycler. As ligands peptide solutions of PIF (wt sequence), PIF_mut-1_, and PIF_mut-3_ at a concentration of 10 μM in buffer containing 10 mM HEPES-Na (pH 7.5) and 100 mM NaCl in a 50-μL volume was mixed with recombinant IDE protein (OriGene ID TP320700) at concentration of 1 μM or with equivalent amounts of DMSO. Final DMSO concentration was maintained at 4% (v/v). The environmentally sensitive dye, SYPRO Orange (Invitrogen, Carlsbad, CA), was used to monitor protein unfolding at a final arbitrary concentration of “6.25X” (the dye is sold as a DMSO stock marked “5000X”). Thermal unfolding was monitored at 0.5°C intervals from 25°C to 85°C. To determine the melting temperature (or midpoint temperature of transition), Tm, for the protein, a Boltzmann model was used to fit protein unfolding curves (thermal melting profiles) (i.e., fluorescence intensity versus temperature) [42] using GraphPad Prism version 3.0. The raw data were transformed, fitted and normalized to have same lower and higher values, and the midpoint temperature of transition Tm was obtained and separately charted as Tm vs ligand type or ligand concentration, where more than one ligand concentrations were tested.

### Chemicals, plasmids and antibodies

We purchased N-Ethylmaleimide (NEM) BioXtra, ≥98% (HPLC) from Sigma-Aldrich (ID 24894398). PIF (MVRIKPGSANKPSDD) was provided by Bio-Synthesis, Inc. (Lewisville, TX, USA). We purchased plasmids containing amyloid precursor protein (APP) from Addgene (pCAX APP 695 #30137). Anti-IDE antibody (SAB2500529) was purchased from Sigma-Aldrich, anti-APP antibody (ALS16390) from Abgent and anti-Aβ17-24 antibody (SIG-39220) from BioLegend.

### Culture of mouse neuroblastoma cell line neuro2a (N2a)

Mouse neuroblastoma cell line neuro2a (N2a) from ATCC was used as previously described [23]. Briefly, the cells were expanded in Dulbecco's Modified EaglesMedium (DMEM; GIBCO Invitrogen), containing 4.5g/Lglucose (GIBCO, Auckland, New Zealand), supplemented with 10% Fetal Bovine Serum (FBS; Sigma S. Louis, USA), 100 U/ml penicillin and 100 μg/ml streptomycin. We kept the cells in a 5% CO_2_ and 95% air atmosphere at 37^°^C. We used Trypsin-EDTA (0.05%; Thermo Fisher Scientific) to detach N2a cells from culture plates. We transfected N2a cells in a 48- well plate scale in complete medium without antibiotics at a density of ~8×10^4^ per well. To prepare plasmid DNA APP-N2a transfection solution for each well, we mixed 1 μg of empty vector or plasmid APP with 25μl OPTI-MEM by gentle pipetting. In parallel, we mixed 1 μl Lipofectamine 2000 with 25μl OPTI-MEM. Following 5 min of incubation at room temperature (RT), we mixed the two by gentle pipetting and incubated for 20 to 30 min at RT to allow DNA/lipid complexes to form. At the end of incubation, we used the 50μl transfection solution to re-suspend the cell pellet. After incubation at RT for 10 min, we added regular growth medium to reach 500 μl of volume and we transferred the cell suspension to the culture plate. After 24-h incubation at 37°C in 5% CO_2_, we replaced the medium with fresh growth medium containing NEM 5 μM, or NEM and PIF 300 nM, (or PBS) respectively, followed by incubation for 48h. Proteins were then extracted and analyzed.

### Protein isolation and western blotting

We isolated proteins using the QIAshredder and the Allprep DNA/RNA/Protein Mini Kits (Qiagen) according to the manufacturer's protocol. Total protein concentration was measured by the bicinchoninic acid protein assay kit (Sigma-Aldrich). We separated protein by SDS-PAGE and transferred onto nitrocellulose membranes (Thermo Fisher Scientific), blocked with 5% BSA and analyzed with a Anti-IDE (1:500), anti-Aβ17-24 (1:300) and anti-APP (1:300). We used HRP-coupled donkey as secondary antibodies (1:1'000; GE Healthcare Life Sciences). We detected binding by chemiluminescence using Amersham ECL Prime Western blotting reagent (GE Healthcare Life Sciences) on a Chemidoc XRS+ system from Bio-Rad Laboratories GmbH.

### Statistical analysis

We used ImageJ software for pixel summation of individual bands. We corrected pixel intensities for background. We standardized protein intensities standardized to the corresponding β-actin bands. Theeffects of PIF in neuronal cells were analyzed by ANOVA followed by two tail t test setting significance at P<0.05.

## SUPPLEMENTARY MATERIALS FIGURES AND TABLES





## References

[1] Masters CL, Multhaup G, Simms G, Pottgiesser J, Martins RN, Beyreuther K (1985). Neuronal origin of a cerebral amyloid: neurofibrillary tangles of Alzheimer's disease contain the same protein as the amyloid of plaque cores and blood vessels. EMBO J.

[2] Pivovarova O, Höhn A, Grune T, Pfeiffer AF, Rudovich N (2016). Insulin-degrading enzyme: new therapeutic target for diabetes and Alzheimer's disease?. Ann Med.

[3] Ruthirakuhan M, Herrmann N, Suridjan I, Abraham EH, Farber I, Lanctôt KL (2016). Beyond immunotherapy: new approaches for disease modifying treatments for early Alzheimer's disease. Expert Opin Pharmacother.

[4] Shen Y, Joachimiak A, Rosner MR, Tang WJ (2006). Structures of human insulin-degrading enzyme reveal a new substrate recognition mechanism. Nature.

[5] Sladek R, Rocheleau G, Rung J, Dina C, Shen L, Serre D, Boutin P, Vincent D, Belisle A, Hadjadj S, Balkau B, Heude B, Charpentier G (2007). A genome-wide association study identifies novel risk loci for type 2 diabetes. Nature.

[6] Qiu WQ, Folstein MF (2006). Insulin, insulin-degrading enzyme and amyloid-beta peptide in Alzheimer's disease: review and hypothesis. Neurobiol Aging.

[7] Cook DG, Leverenz JB, McMillan PJ, Kulstad JJ, Ericksen S, Roth RA, Schellenberg GD, Jin LW, Kovacina KS, Craft S (2003). Reduced hippocampal insulin-degrading enzyme in late-onset Alzheimer's disease is associated with the apolipoprotein E-epsilon4 allele. Am J Pathol.

[8] Rudovich N, Pivovarova O, Fisher E, Fischer-Rosinsky A, Spranger J, Möhlig M, Schulze MB, Boeing H, Pfeiffer AF (2009). Polymorphisms within insulin-degrading enzyme (IDE) gene determine insulin metabolism and risk of type 2 diabetes. J Mol Med (Berl).

[9] Maianti JP, McFedries A, Foda ZH, Kleiner RE, Du XQ, Leissring MA, Tang WJ, Charron MJ, Seeliger MA, Saghatelian A, Liu DR (2014). Anti-diabetic activity of insulin-degrading enzyme inhibitors mediated by multiple hormones. Nature.

[10] Vekrellis K, Ye Z, Qiu WQ, Walsh D, Hartley D, Chesneau V, Rosner MR, Selkoe DJ (2000). Neurons regulate extracellular levels of amyloid beta-protein via proteolysis by insulin-degrading enzyme. J Neurosci.

[11] Carrasquillo MM, Belbin O, Zou F, Allen M, Ertekin-Taner N, Ansari M, Wilcox SL, Kashino MR, Ma L, Younkin LH, Younkin SG, Younkin CS, Dincman TA (2010). Concordant association of insulin degrading enzyme gene (IDE) variants with IDE mRNA, Abeta, and Alzheimer's disease. PLoS One.

[12] Zuo X, Jia J (2009). Promoter polymorphisms which modulate insulin degrading enzyme expression may increase susceptibility to Alzheimer's disease. Brain Res.

[13] Barnea ER (2004). Insight into early pregnancy events: the emerging role of the embryo. Am J Reprod Immunol.

[14] Barnea ER, Kirk D, Ramu S, Rivnay B, Roussev R, Paidas MJ (2012). PreImplantation Factor (PIF) orchestrates systemic antiinflammatory response by immune cells: effect on peripheral blood mononuclear cells. Am J Obstet Gynecol.

[15] Stamatkin CW, Roussev RG, Stout M, Absalon-Medina V, Ramu S, Goodman C, Coulam CB, Gilbert RO, Godke RA, Barnea ER (2011). PreImplantation Factor (PIF) correlates with early mammalian embryo development-bovine and murine models. Reprod Biol Endocrinol.

[16] Ramu S, Stamatkin C, Timms L, Ruble M, Roussev RG, Barnea ER (2013). PreImplantation factor (PIF) detection in maternal circulation in early pregnancy correlates with live birth (bovine model). Reprod Biol Endocrinol.

[17] Duzyj CM, Barnea ER, Li M, Huang SJ, Krikun G, Paidas MJ (2010). Preimplantation factor promotes first trimester trophoblast invasion. Am J Obstet Gynecol.

[18] Roussev RG, Dons'koi BV, Stamatkin C, Ramu S, Chernyshov VP, Coulam CB, Barnea ER (2013). Preimplantation factor inhibits circulating natural killer cell cytotoxicity and reduces CD69 expression: implications for recurrent pregnancy loss therapy. Reprod Biomed Online.

[19] Paidas MJ, Krikun G, Huang SJ, Jones R, Romano M, Annunziato J, Barnea ER (2010). A genomic and proteomic investigation of the impact of preimplantation factor on human decidual cells. Am J Obstet Gynecol.

[20] Weiss L, Or R, Jones RC, Amunugama R, JeBailey L, Ramu S, Bernstein SA, Yekhtin Z, Almogi-Hazan O, Shainer R, Reibstein I, Vortmeyer AO, Paidas MJ (2012). Preimplantation factor (PIF*) reverses neuroinflammation while promoting neural repair in EAE model. J Neurol Sci.

[21] Migliara G, Mueller M, Piermattei A, Brodie C, Paidas MJ, Barnea ER, Ria F (2017). PIF* promotes brain re-myelination locally while regulating systemic inflammation- clinically relevant multiple sclerosis M.smegmatis model. Oncotarget.

[22] Mueller M, Zhou J, Yang L, Gao Y, Wu F, Schoeberlein A, Surbek D, Barnea ER, Paidas M, Huang Y (2014). PreImplantation factor promotes neuroprotection by targeting microRNA let-7. Proc Natl Acad Sci USA.

[23] Mueller M, Schoeberlein A, Zhou J, Joerger-Messerli M, Oppliger B, Reinhart U, Bordey A, Surbek D, Barnea ER, Huang Y, Paidas M (2015). PreImplantation Factor bolsters neuroprotection via modulating Protein Kinase A and Protein Kinase C signaling. Cell Death Differ.

[24] Barnea ER, Almogi-Hazan O, Or R, Mueller M, Ria F, Weiss L, Paidas MJ (2015). Immune regulatory and neuroprotective properties of preimplantation factor: from newborn to adult. Pharmacol Ther.

[25] Weiss L, Bernstein S, Jones R, Amunugama R, Krizman D, Jebailey L, Almogi-Hazan O, Yekhtin Z, Shiner R, Reibstein I, Triche E, Slavin S, Or R, Barnea ER (2011). Preimplantation factor (PIF) analog prevents type I diabetes mellitus (TIDM) development by preserving pancreatic function in NOD mice. Endocrine.

[26] Chen YC, Rivera J, Fitzgerald M, Hausding C, Ying YL, Wang X, Todorova K, Hayrabedyan S, Barnea ER, Peter K (2016). PreImplantation factor prevents atherosclerosis via its immunomodulatory effects without affecting serum lipids. Thromb Haemost.

[27] Azar Y, Shainer R, Almogi-Hazan O, Bringer R, Compton SR, Paidas MJ, Barnea ER, Or R (2013). Preimplantation factor reduces graft-versus-host disease by regulating immune response and lowering oxidative stress (murine model). Biol Blood Marrow Transplant.

[28] Shainer R, Almogi-Hazan O, Berger A, Hinden L, Mueller M, Brodie C, Simillion C, Paidas M, Barnea ER, Or R (2016). PreImplantation factor (PIF) therapy provides comprehensive protection against radiation induced pathologies. Oncotarget.

[29] Vingtdeux V, Chandakkar P, Zhao H, Blanc L, Ruiz S, Marambaud P (2015). CALHM1 ion channel elicits amyloid-β clearance by insulin-degrading enzyme in cell lines and in vivo in the mouse brain. J Cell Sci.

[30] Barnea ER, Lubman DM, Liu YH, Absalon-Medina V, Hayrabedyan S, Todorova K, Gilbert RO, Guingab J, Barder TJ (2014). Insight into PreImplantation Factor (PIF*) mechanism for embryo protection and development: target oxidative stress and protein misfolding (PDI and HSP) through essential RIKP [corrected] binding site. PLoS One.

[31] Hersh LB (2006). The insulysin (insulin degrading enzyme) enigma. Cell Mol Life Sci.

[32] Malito E, Ralat LA, Manolopoulou M, Tsay JL, Wadlington NL, Tang WJ (2008). Molecular bases for the recognition of short peptide substrates and cysteine-directed modifications of human insulin-degrading enzyme. Biochemistry.

[33] Im H, Manolopoulou M, Malito E, Shen Y, Zhao J, Neant-Fery M, Sun CY, Meredith SC, Sisodia SS, Leissring MA, Tang WJ (2007). Structure of substrate-free human insulin-degrading enzyme (IDE) and biophysical analysis of ATP-induced conformational switch of IDE. J Biol Chem.

[34] Niesen FH, Berglund H, Vedadi M (2007). The use of differential scanning fluorimetry to detect ligand interactions that promote protein stability. Nat Protoc.

[35] O'Brien C, Barnea ER, Martin P, Levy C, Sharabi E, Bhamidimarri K, Martin K, Arosemena L, Schiff ER (2018). Randomized, Double-Blind, Placebo-Controlled, Single Ascending Dose Trial of Synthetic Preimplantation Factor in Autoimmune Hepatitis. Hepatology Communications.

[36] Zemla A (2003). LGA: A method for finding 3D similarities in protein structures. Nucleic Acids Res.

[37] Zemla A, Geisbrecht B, Smith J, Lam M, Kirkpatrick B, Wagner M, Slezak T, Zhou CE (2007). STRALCP—structure alignment-based clustering of proteins. Nucleic Acids Res.

[38] Barnea ER, Kirk D, Todorova K, McElhinney J, Hayrabedyan S, Fernández N (2015). PIF direct immune regulation: blocks mitogen-activated PBMCs proliferation, promotes TH2/TH1 bias, independent of Ca(2+). Immunobiology.

[39] London N, Raveh B, Cohen E, Fathi G, Schueler-Furman O (2011). Rosetta FlexPepDock web server—high resolution modeling of peptide-protein interactions. Nucleic Acids Res.

[40] Shatsky M, Nussinov R, Wolfson HJ (2004). A method for simultaneous alignment of multiple protein structures. Proteins.

[41] Kurcinski M, Jamroz M, Blaszczyk M, Kolinski A, Kmiecik S (2015). CABS-dock web server for the flexible docking of peptides to proteins without prior knowledge of the binding site. Nucleic Acids Res.

[42] Ericsson UB, Hallberg BM, Detitta GT, Dekker N, Nordlund P (2006). Thermofluor-based high-throughput stability optimization of proteins for structural studies. Anal Biochem.

