# Supplementary material for: The core sequence of PIF competes for insulin/amyloid β in insulin degrading enzyme: potential treatment for Alzheimer's disease
Source: Oncotarget. 2018 Sep 21;9(74):33884–95. doi: 10.18632/oncotarget.26057 (PMC6188057; doi:10.18632/oncotarget.26057)
Supplement: Supplementary file 1 [file oncotarget-09-33884-s001.pdf]

The core sequence of PIF competes for insulin/amyloid β in insulin degrading enzyme: potential treatment for Alzheimer's disease

SUPPLEMENTARY MATERIALS

Supplementary Table 1: Excel Tables summarizing PepSite2 prediction of PIF peptide:protein binding interface. PepSite2 putative PIF:target binding residues interaction of ProtoArray® true positive and negative interactors was queried using a TAVERNA workbench operated REST service. The query scored about 200 PDBs corresponding to positive hits and more than 2500 PDBs corresponding to negative hits. The statistical probability of important residues was determined, resulting in M*RIKP**** to be most likely binding pattern.

See Supplementary File 1

PIF target	PDB	Highest ΔE [kcal/mol]	PIF_{1-15} residues and target residues positions														
			M	V	R	I	K	P	G	S	A	N	K	P	S	D	D
Substrate free IDE – closed conformation	2JG4:A	3.03				G D E A S K											
Substrate free IDE – closed conformation	2JG4:B	2.04					G D A										
IDE bound to Insulin	2WBY:A	3.78				G G E D S											

Supplementary Figure 1: *In Silico* Mutagenesis of PIF-IDE docking. Using an *in silico* mutagenesis approach (BeAtMuSiC), we mutated the PIF residues at its binding interfaces in several FlexPepDock docking models of PIF bound to IDE in closed or opened conformation. We selected top ranking models with supposedly decreased affinity of binding, determined by differences (increase) in ligand-receptor model Energy [kCal/mol]. Based on those results we generated two mutants, designated as "$\mathbf{PIF_{mut1}}$" and "$\mathbf{PIF_{mut3}}$" with sequences "MVRIK**E**GSANKPSDD" and "MVR**G**KPGSANKPSDD", respectively. The $\mathbf{PIF_{mut1}}$ was selected to be non-IDE specific, while $\mathbf{PIF_{mut3}}$ was selected to be among the top-ranking IDE specific mutants.
